# Osteoarthritis: Role of Peroxisome Proliferator-Activated Receptors

**DOI:** 10.3390/ijms241713137

**Published:** 2023-08-24

**Authors:** Weibei Sheng, Qichang Wang, Haotian Qin, Siyang Cao, Yihao Wei, Jian Weng, Fei Yu, Hui Zeng

**Affiliations:** 1 National & Local Joint Engineering Research Center of Orthopaedic Biomaterials, Peking University Shenzhen Hospital, Shenzhen 518036, China; 2 Department of Bone & Joint Surgery, Peking University Shenzhen Hospital, Shenzhen 518036, China

**Keywords:** peroxisome proliferator-activated receptors, PPAR, osteoarthritis, chondrocytes

## Abstract

Osteoarthritis (OA) represents the foremost degenerative joint disease observed in a clinical context. The escalating issue of population aging significantly exacerbates the prevalence of OA, thereby imposing an immense annual economic burden on societies worldwide. The current therapeutic landscape falls short in offering reliable pharmaceutical interventions and efficient treatment methodologies to tackle this growing problem. However, the scientific community continues to dedicate significant efforts towards advancing OA treatment research. Contemporary studies have discovered that the progression of OA may be slowed through the strategic influence on peroxisome proliferator-activated receptors (PPARs). PPARs are ligand-activated receptors within the nuclear hormone receptor family. The three distinctive subtypes—PPARα, PPARβ/δ, and PPARγ—find expression across a broad range of cellular terminals, thus managing a multitude of intracellular metabolic operations. The activation of PPARγ and PPARα has been shown to efficaciously modulate the NF-κB signaling pathway, AP-1, and other oxidative stress-responsive signaling conduits, leading to the inhibition of inflammatory responses. Furthermore, the activation of PPARγ and PPARα may confer protection to chondrocytes by exerting control over its autophagic behavior. In summation, both PPARγ and PPARα have emerged as promising potential targets for the development of effective OA treatments.

## 1. Introduction

Osteoarthritis (OA) represents a pervasive degenerative disease with a multifactorial etiology encompassing elements such as injury, genetic predisposition, obesity, age, and gender [1,2,3]. A defining characteristic of OA involves the structural degradation of chondrocytes in articular cartilage. As a tissue devoid of blood vessels, the homeostatic maintenance of the cartilage matrix rests solely upon chondrocytes. Consequently, fragmentary cartilage, resulting from wear and tear, instigates inflammation within the synovium.

**Figure 1:**
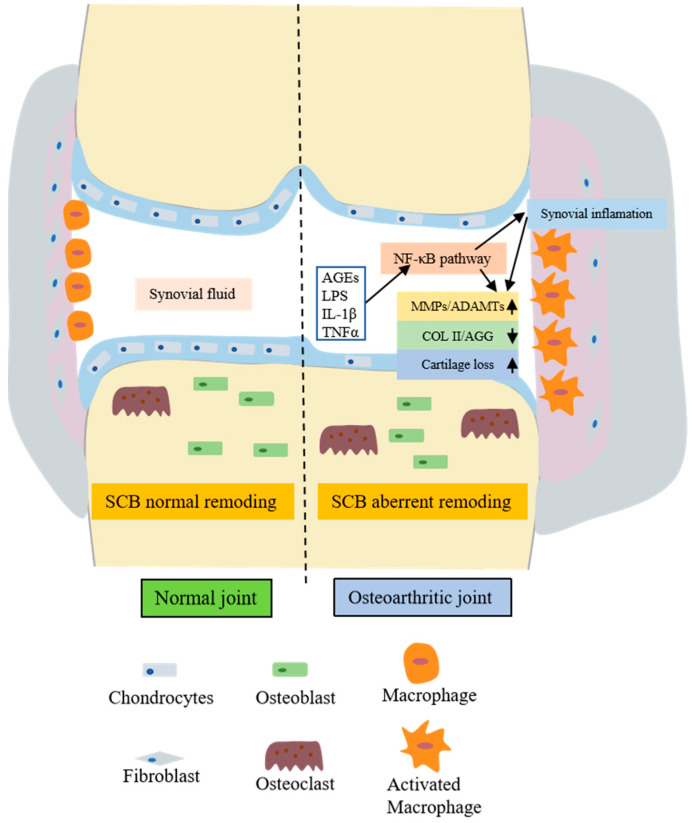
Comparative schematic structures of normal and osteoarthritic joints. The figure displays multiple factors that contribute to chondrocyte catabolism and ECM degradation in osteoarthritic joints. Inflammatory agents such as AGEs, LPS, IL-1β, and TNF-α can induce synovial inflammation by influencing the NF-κB pathway. These inflammatory mediators enhance the expression of MMPs and ADAMTs via the NF-κB pathway, leading to the downregulation of COL II and AGG and consequently causing the loss of the cartilage matrix. Under the influence of inflammatory agents, various cells in the synovium, including macrophages and osteoclasts, are activated, intensifying the inflammatory response in the joint and ultimately precipitating the onset of OA. ECM, extracellular matrix; AGEs, advanced glycation end products; LPS, lipopolysaccharides; IL-1β, interleukin-1 beta; TNF-α, tumor necrosis factor alpha; NF-κB, nuclear factor kappa B; MMPs, matrix metalloproteinases; ADAMTs, a disintegrin and metalloproteinase with thrombospondin motifs; COL II, collagen type II; AGG, aggrecan; OA, Osteoarthritis.

Inflammatory mediators produced by the synovium, such as interleukin-1β (IL-1β), the tumor necrosis factor α (TNF-α), nitric oxide (NO), and prostaglandin E2 (PGE2), demonstrate significant upregulation within the synovium of OA-afflicted joints. These inflammatory factors inhibit the primary organic components of the cartilage matrix—type II collagen and aggregated proteoglycans. Furthermore, they stimulate chondrocytes to generate enzymes that degrade the cartilage matrix, thus promoting chondrocyte catabolism [4]. The production of NO can also foster cartilage degradation and apoptosis. Figure 1 illustrates a schematic representation of both a normal and an OA joint.

To manage OA, the prevailing treatment regimen involves the prescription of nonsteroidal anti-inflammatory drugs (NSAIDs), opioids, and/or intraarticular corticosteroids. While these therapeutic agents employ varying mechanisms of action, they all strive towards the reduction in inflammation and/or pain, alleviation of symptoms, and enhancement of patient quality of life. However, the long-term application of NSAIDs is accompanied by an array of adverse effects including gastrointestinal ulcers, renal failure, and the inhibition of vital physiological functions [5]. Therefore, the search for safer and more efficacious OA treatments remains a pressing necessity.

As research in the field deepens, increasing evidence points towards the potential improvement of cartilage function and the resistance and retardation of OA progression through the interference with members of the nuclear receptor superfamily, specifically, the peroxisome proliferator-activated receptors (PPARs) [6,7]. PPARs, as ligand-activated transcription factors, play a pivotal role in lipid homeostasis. There are three subtypes of PPAR: α, γ, and β/δ [8,9]. A multitude of studies indicate that targeting PPARγ and PPARα can modulate the inflammatory response [10]. Both PPARγ and PPARα can inhibit the expression of inflammatory genes, including cytokines, metalloproteinases, and acute phase proteins [11,12]. Additionally, PPARα/γ can regulate oxidative stress-sensitive signaling pathways, such as redox-responsive nuclear factor-κB, activator protein-1, and signal transducer proteins [13,14]. These findings propose that PPARα/γ could function as potential therapeutic targets for inflammation-associated diseases. This article delves into the relationship between PPARα/β and OA and evaluates the protective effect of PPARα/γ on OA.

## 2. Overview of PPAR

### 2.1. Family of PPAR

The peroxisome proliferator-activated receptor (PPAR) is a member of the nuclear receptor superfamily and functions as a ligand-activated transcription factor. PPAR is further divided into three distinct subtypes: PPARα, PPARγ, and PPARβ/δ. The human gene for PPARα is situated on chromosome 22, while PPARγ—which has been identified to possess two isoforms [15]—is located on chromosome 3, and PPARβ/δ resides on chromosome 6 [16,17,18].

Analogous to other members of the superfamily, PPAR possesses six regions (A–F), encompassing three primary functional domains: an N-terminal domain (NTD) housing the constitutive activation function 1 (AF-1), two DNA-binding domains (DBD) containing zinc finger motifs, and a ligand-binding domain (LBD) with the ligand-dependent AF-2 subunit at its C-terminus [19,20].

**Figure 2:**
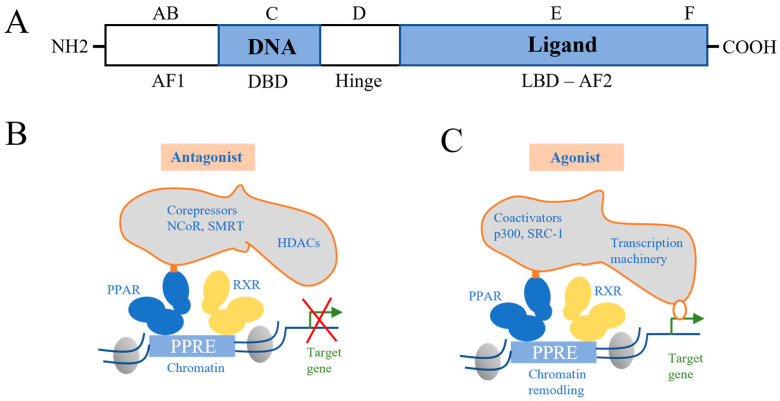
Modes of action of PPARs. (**A**) Structural and functional domain organization of nuclear receptors. (**B**) Antagonist-activated PPAR forms a PPAR: RXR heterodimer that binds to a corepressor and binds to histone deacetylase (HDAC), which maintains a low acetylation state at the tail of the histone and inhibits gene expression when binding to PPRE. (**C**) The binding of agonists triggers the clearance of corepressors, thereby achieving effective recruitment of co-activators and transcriptional activation.

Transcriptional regulation by PPAR proceeds via heterodimerization with the nuclear receptor retinoid X receptor (RXR) [21,22]. Upon activation of the PPAR:RXR heterodimer, conformational changes transpire, leading to the liberation of co-repressors (e.g., SMRT and NCoR) and the enlistment of co-activators (e.g., p300, SRC-1). The PPAR:RXR heterodimer associates with the peroxisome proliferator response element (PPRE) within the regulatory region of its target genes [23]. Figure 2 encapsulates the structure of PPAR.

PPARα is ubiquitously present in a variety of cells, including endothelial cells, hepatocytes, and chondrocytes [24,25,26,27]. PPARγ expresses as two isoforms, γ1 and γ2, differentiated at their N terminus. PPARγ2 is predominantly expressed in adipose tissues [28,29,30], whereas PPARγ1 displays a broader expression pattern, extending to domains such as the gut, brain, vascular cells, and specific types of immune and inflammatory cells [31,32]. PPARδ is globally expressed across bodily tissues and regulates energy expenditure in cells [33,34].

### 2.2. PPAR Ligands

The natural ligands for PPAR comprise lipid-derived metabolites such as fatty acids, acyl-CoAs, and glycerol-phospholipids [35]. Given that PPARs serve as crucial regulators of energy homeostasis and inflammation, a substantial body of research has focused on their synthetic ligands. Fibrates have been demonstrated to selectively activate PPARα, while thiazolidinediones have been shown to activate PPARγ [36]. A summary of common PPAR ligands is provided in Table 1.

### 2.3. Function of PPAR

PPARs assume a critical role in governing energy homeostasis and inflammation. More specifically, PPARα primarily partakes in processes such as adipocyte differentiation, cholesterol metabolism, and the inflammatory response. PPARγ can modulate inflammation and immunity, regulate tumorigenesis, and exhibits a potent capacity to manage adipose differentiation. PPARβ/δ primarily oversees the homeostasis of lipids, glucose, and energy [36].

## 3. The Role of PPARα/γ in OA Chondrocytes/Cartilage

**Figure 3:**
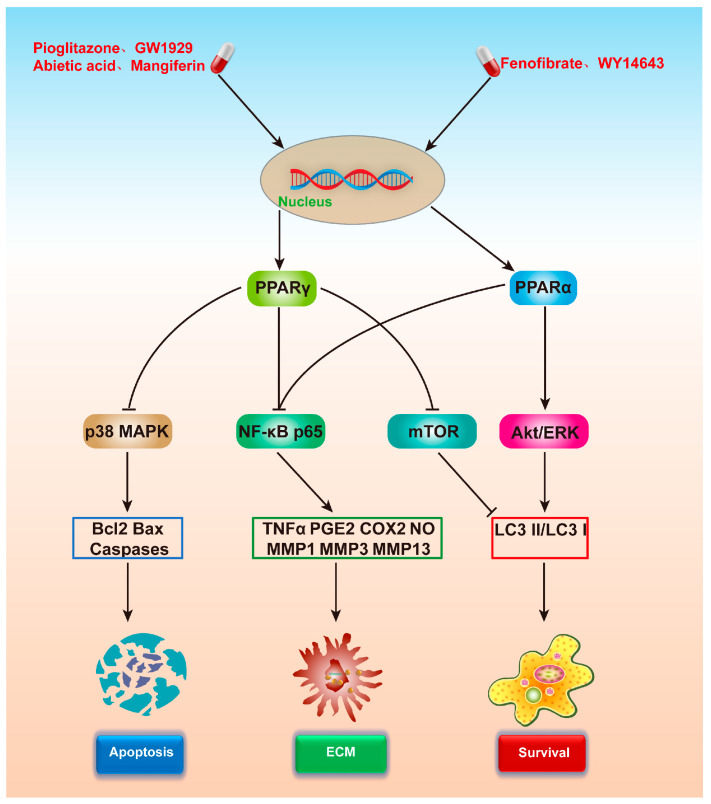
Mechanism of chondrocyte protection following PPARα/γ activation. PPARγ can stimulate autophagy by inhibiting the p38 MAPK pathway, suppress the NF-κB pathway to foster extracellular matrix synthesis, and inhibit the mammalian target of the rapamycin (mTOR) pathway to regulate cell activity. PPARα can foster the synthesis of the extracellular matrix via the NF-κB pathway. PPARα can stimulate cell activity by enhancing the extracellular signal-regulated kinase (ERK) and protein kinase B (Akt) pathways. PPARα/γ, peroxisome proliferator-activated receptor α/γ; MAPK, mitogen-activated protein kinase; mTOR, mammalian target of rapamycin; ERK, extracellular signal-regulated kinase; Akt, protein kinase B.

Articular cartilage degeneration is the principal factor contributing to OA, and studies indicate that PPARα/γ plays a vital role in maintaining articular cartilage homeostasis [37,38]. The impacts of PPARα/γ on chondrocytes are summarized in Figure 3 and Table 2.

### 3.1. Inhibition of Matrix Metalloproteinases (MMPs) in OA Chondrocytes

MMPs can be grouped into five categories. First, collagenases consist of MMP-1, -8, and -13; second, gelatinases include MMP-2 and -9; third, stromelysins consist of MMP-3, -10, and -11, which degrade the collagen matrix proteins; fourth, the membrane-type MMPs specifically consist of MMP-14, -15, -16, -17, -24, and -25; and fifth, other subgroups namely consist of MMP-7, -11, -12, -20, and -23 [49]. Under normal joint conditions, matrix proteases are expressed at minimal levels. However, in inflamed joints, their expression is significantly amplified [50,51]. MMPs are implicated in various diseases, including OA, where they may intensify cartilage degradation, exacerbating OA progression. Therefore, the targeted suppression of MMPs might serve as an efficacious method for alleviating OA.

Of these, MMP-1, MMP-3, MMP-9, and MMP13 play a particularly important role [52,53]. Factors such as pro-inflammatory agents (e.g., IL-1β), advanced glycation end products (AGEs), and high glucose can dramatically augment cartilage catabolism, thereby damaging the cartilage matrix. Activation of PPARα/γ can reduce chondrocyte catabolism under the various stimuli mentioned above, thereby offering protection to chondrocytes. Studies discovered that PPARγ agonists 15d-PGJ2 and GI262570 (10 μM) inhibited IL-1β- and TNF-α-induced proteoglycan degradation [42], an effect associated with the inhibition of MMP-3 and -9 production. Research has shown that the PPARα agonist Wy-14643 reduced the mRNA expression of MMP-1, MMP-3, and MMP-13 in cartilage explants responding to IL-1β [39]. Further studies by Chen et al., Ma et al., and Zhang et al. demonstrated that the PPARγ agonist pioglitazone inhibited AGEs-induced MMP-13 expression by antagonizing NF-κB activation in chondrocytes [43,45,46]. The role of PPARγ in mitigating the hyperglycemia-induced catabolic response and collagen degradation in human chondrocytes has also been proposed [44]. In conclusion, PPARs play a pivotal role in influencing the effects of MMPs.

### 3.2. Inhibition of Inflammatory-Related Factors in OA Cartilage

Inflammatory mediators such as NO and PGE2 play a crucial role in OA pathogenesis. NO contributes to OA progression through its catabolic role, mediating inflammation, inhibiting collagen and proteoglycan synthesis, and promoting apoptosis [54]. PGE2 is the most abundant prostaglandin in humans and is a primary inflammatory mediator in inflammatory diseases such as OA and rheumatoid arthritis [55,56]. Upregulation of IL-1β and TNF-α in chondrocytes can trigger the production of inflammatory mediators such as NO and PGE2. Reducing these inflammatory mediators in OA cartilage can effectively mitigate OA progression. The targeted activation of PPARα/γ can inhibit various inflammatory mediators in chondrocytes. Clockaerts et al. found that the PPARα agonist Wy-14643 reduced the secretion of the inflammatory marker NO in IL-1β-treated cartilage explants [39]. The PPARγ agonist pioglitazone can inhibit AGEs-induced chondrocyte inflammatory response [43,46].

### 3.3. Inhibition of Chondrocyte Apoptosis

Factors such as IL-1β and AGEs can induce chondrocyte apoptosis. The targeted activation of PPARγ can reduce IL-1β/AGEs-induced chondrocyte apoptosis. Activation of PPARγ can substantially inhibit the IL-1β-induced expressions of COX-2 and PGE2 in chondrocytes, thereby reducing chondrocyte apoptosis and slowing down the pathological progression of OA [48]. Additionally, PPARγ agonists can inhibit the expression of MMP-13 and the apoptosis of chondrocytes by suppressing AGEs [45].

### 3.4. Regulating the Autophagic Activity of Chondrocytes

PPARs exert an influence on the physiological processes of chondrocytes prominently through their regulation of autophagy. Autophagy serves as an adaptive response that equips cells with the capacity to withstand stressors [57,58]. It denotes a process where cytoplasmic proteins or organelles are ensnared and encased within vesicles, which later conjoin with lysosomes to form autolysosomes that dismantle the substances within the encapsulated lysosomes. On the basis of cargo sequestration mechanisms, autophagy is classified into three distinct types: microautophagy, chaperone-mediated autophagy, and macroautophagy [59].

Autophagy in chondrocytes is pivotal in the onset of OA, instigated by factors such as aging and trauma. A decline in autophagy levels culminates in damage to the organelles and the aggregation of macromolecules within cells, which compromises the viability of chondrocytes and subsequently contributes to age-related OA [60]. Autophagy-associated proteins (namely LC3-II, a marker for mammalian autophagosomes) seem to diminish in mechanically damaged cartilage [61,62]. By stimulating the autophagic activity of chondrocytes pharmacologically, chondrocytes can be effectively safeguarded [61,62]. Our research revealed that PPARα activation via Wy-14643 augments proteoglycan synthesis by enhancing autophagy in lipopolysaccharide (LPS)-treated chondrocytes. Further, the intra-articular administration of Wy-14643 diminishes the degeneration of articular cartilage, promotes proteoglycan synthesis, and intensifies autophagy in vivo [40]. Novel therapeutic concepts also encompass the activation of PPARγ, the inhibition of the mTOR signaling pathway, and the induction of autophagy in chondrocytes [47].

## 4. Effect of PPARs on OA-Related Signaling Pathways

The targeted activation of PPARα/γ can mitigate chondrocyte inflammation via various signaling pathways, manage its autophagy activity, and repress chondrocyte apoptosis. The signaling pathways most emblematic of OA include NF-κB, Akt/mTOR, and MAPK. This discussion presents a summary of the modulatory impacts of PPARα/γ activating ligands on the NF-κB signaling pathway, the Akt/mTOR signaling pathway, and the MAPK signaling pathway.

### 4.1. NF-κB Signaling Pathway

PPARs may slow down the progression of OA by impacting the NF-κB signaling pathway [63,64,65]. NF-κB is a crucial transcription factor that orchestrates inflammatory responses [66]. Under normal circumstances, it attaches to the inhibitor IκB in the cytoplasm. However, under the influence of stimuli such as cytokines or LPS, IκB undergoes degradation, which enables the translocation of NF-κB p65 to the nucleus, thereby dictating the production of inflammatory mediators such as PGE2, NO, and TNF-α. Given its potential as a target for treating inflammatory diseases such as rheumatoid arthritis and OA, NF-κB attracts significant attention [67].

The role of AGEs in inducing apoptosis in chondrocytes has been elucidated. Furthermore, they degrade the cartilage matrix by elevating the expression of MMP-13. Pioglitazone, a PPARγ agonist, can curb the expression of AGEs, thereby obstructing the degradation of IκB in chondrocytes, the nuclear translocation of NF-κB p65, impeding its expression and ultimately safeguarding chondrocytes [45,46]. Studies have also discovered that pioglitazone can restrain AGEs-induced nuclear p65 expression, consequently reducing the expression of TNF-α and MMP-13 in rabbit chondrocytes, thereby manifesting its anti-inflammatory properties [43]. In conclusion, mediating the NF-κB signaling pathway by modulating PPARs may be a promising therapeutic strategy for OA.

### 4.2. Akt/mTOR Signaling Pathway

PPARs are known to exert an influence on the Akt/mTOR signaling pathway. The mammalian target of rapamycin (mTOR) is a pivotal protein kinase, governing an array of cellular processes inclusive of cell growth, metabolism, survival, and lifespan [40,68]. mTOR forms two complexes, namely mTOR complex 1 (mTORC1) and mTOR complex 2 (mTORC2), in conjunction with raptor and rictor. By phosphorylating and inactivating the 4E-binding protein (4E-BP1) and stimulating S6K1, mTOR orchestrates protein synthesis [69]. Moreover, mTOR thwarts ULK1 activation by phosphorylating and disrupting the interaction between ULK1 and AMPK, subsequently repressing autophagy, a crucial mechanism for cell survival [70,71]. Akt, another integral regulator of mTOR, is generally deemed an inhibitor of autophagy [72,73,74,75]. The utilization of the PPARγ agonist pioglitazone can regulate the Akt/mTOR signaling pathway, thereby maintaining chondrocyte viability and ameliorating the inflammatory response via the induction of chondrocyte autophagy [47]. Additionally, the activation of PPARγ coupled with the inhibition of the mTOR signaling pathway initiates autophagy in chondrocytes, thereby enhancing their activity [76]. The PPARα agonist Wy-14643 is another intervention found to promote proteoglycan synthesis via amplifying autophagy and augmenting Akt phosphorylation in OA chondrocytes [76]. These results suggest that the targeted activation of PPARα/γ can boost chondrocyte activity via the modulation of the Akt/mTOR signaling pathway and the induction of autophagy. To conclude, the Akt/mTOR signaling pathway constitutes a crucial node for PPARs’ involvement in inflammation, offering guidance for OA treatment.

### 4.3. MAPK Signaling Pathway

Numerous studies have uncovered that PPARs exert a substantial regulatory effect on MAPK [77,78]. MAPK signaling plays a critical role in diverse cellular processes such as cell proliferation, differentiation, and apoptosis, and its pathway primarily comprises p38, JNK, and ERK. It was demonstrated that chondrocyte apoptosis correlated with an increased phosphorylation of p38 MAPK [79]. The PPARγ agonist GW1929 can suppress NOX2/ROS/p38MAPK activation and mitigate IL-1β-induced chondrocyte apoptosis in OA [44]. Additionally, pioglitazone, another PPARγ agonist, can inhibit p38 MAPK activation and curb AGEs-induced apoptosis [45].

The ERK1/2 signaling pathway forms a central MAPK cascade that regulates cellular processes such as proliferation, differentiation, development, learning, survival, and apoptosis, among others, under certain conditions [80]. Studies have indicated that activating the ERK1/2 signaling pathway can stimulate chondrocyte autophagy, thereby augmenting chondrocyte activity. Anti-inflammatory constituents in some traditional Chinese medicines can effectively activate ERK1/2 and protect chondrocytes. It has been reported that curcumin and angelica polysaccharides can induce autophagy and inhibit chondrocyte apoptosis via the ERK1/2 pathway [81,82]. The PPARα agonist Wy-14643 can stimulate the ERK1/2 signaling pathway and induce autophagy in chondrocytes, thereby protecting cartilage [40]. PPARs maintain a close association with the MAPK signaling pathway, which has emerged as a major research focus in recent years.

## 5. Active Small Molecule Drugs Targeting Activation of PPARγ

PPARγ is currently a prominent focus in inflammation research. Studies have identified several compounds including abietic acid, astragalin, betulinic acid, mangiferin, oridonin, and losartan, which can activate PPARγ in chondrocytes and notably inhibit IL-1β-induced inflammatory responses [83,84,85,86,87,88]. A summarization of the effects of these active molecules on OA chondrocytes can be found in Table 3.

### 5.1. Abietic Acid

Abietic acid is a tricyclic diterpenoid compound and is one of the most important resin acids, known for its anti-inflammatory and antioxidant properties [91]. Studies demonstrate that abietic acid can significantly diminish the expression of TNF-α, NO, PGE2, and COX-2 in human OA chondrocytes, reduce the IL-1β-induced expression of MMP-1, MMP-3, and MMP-13, and enhance chondrocyte function. In addition, it has been observed that the inhibition of PPARγ can mitigate the inhibitory effect of abietic acid on TNF-α, NO, and PGE2 [83]. These findings suggest that the anti-inflammatory properties of abietic acid are primarily facilitated via its interaction with PPARγ.

### 5.2. Astragalin

Astragalin, a bioactive compound isolated from roses, has been reported to exhibit various pharmacological properties, including anti-inflammatory and antioxidant effects [92,93]. Astragalin can inhibit the expression of PGE2, iNOS, and COX-2 in human OA chondrocytes. When PPARγ is antagonized, the astragalin-mediated inhibition of IL-1β-induced NO and PGE2 production is attenuated [84]. These findings suggest that astragalin inhibits IL-1β-induced inflammatory mediators by activating PPARγ in human OA chondrocytes.

### 5.3. Betulinic Acid

Betulinic acid (BA), a triterpenoid isolated from birch bark, is reported to possess anti-inflammatory, antitumor, and antiviral properties [94,95]. BA inhibits IL-1β-induced production of MMPs, PGE2, and NO in human OA chondrocytes and suppresses IL-1β-induced NF-κB activation. Studies have demonstrated an activating effect of BA on PPARγ, and it has been found that PPARγ antagonism nullifies the inhibitory effect of BA on PGE2 and NO [85]. This evidence indicates that BA could suppress IL-1β-induced inflammation in OA chondrocytes by activating PPARγ.

### 5.4. Mangiferin

Mangiferin (MFN), a flavonoid glucoside found in botanical medicines and foods, has been reported to have anti-inflammatory properties. MFN inhibits IL-1β-induced production of inflammatory mediators PGE2 and NO in human OA chondrocytes, and it suppresses IL-1β-induced production of MMP-1 and MMP-3, as well as NF-κB activation. The PPARγ inhibitor, GW9662, significantly reverses the anti-inflammatory effect of MFN [86]. These findings suggest that PPARγ plays a crucial role in the anti-inflammatory action of MFN in chondrocytes.

### 5.5. Oridonin

Oridonin, a diterpenoid compound isolated from *Rabdosia rubescens*, possesses anti-inflammatory properties [96]. It significantly inhibits the expression of MMPs, COX-2, PGE2, and iNOS in human OA chondrocytes. Research suggests that it exerts its anti-inflammatory effects primarily by inhibiting the NF-κB signaling pathway. Additionally, oridonin can upregulate the expression of PPARγ in chondrocytes in a concentration-dependent manner, and PPARγ antagonists can reverse the anti-inflammatory effect of oridonin [87]. These findings suggest that oridonin reduces pro-inflammatory factors and catabolism in chondrocytes by activating PPARγ.

### 5.6. Losartan

Losartan, an antagonist of the angiotensin II receptor, has been shown to slow the progression of OA [97]. Losartan can decrease the expression of pro-inflammatory factors IL-1β, IL-6, TNF-α, and the catabolic factors MMP-13, ADAMTS4, ADAMTS5, COX-2 and TGF-β1 in OA chondrocytes. The inhibition of these factors can effectively protect cartilage and alleviate the progression of OA. Losartan can also enhance the expression of PPARγ in OA chondrocytes. The inhibitory effect of losartan on inflammation and catabolism is weakened when siRNA is used to knock down the expression of PPARγ in chondrocytes [88]. These results indicate that PPARγ plays a crucial role in the anti-inflammatory and anti-catabolic actions of losartan in OA chondrocytes.

## 6. Adjusting PPARγ Promoter Methylation in Chondrocytes to Alleviate OA

PPAR’s cellular influence is multifaceted, spanning from receptor activation and pathway intervention to epigenetic modification. Among the various epigenetic modifications, such as DNA methylation, histone acetylation, and miRNA interference, DNA methylation emerges as the most extensively studied regulatory mechanism impacting OA development [98]. DNA methylation is catalyzed by three biologically active DNA methyltransferases (DNMTs)—DNMT1, DNMT3a, and DNMT3b—which append a methyl group to the fifth carbon of a cytosine residue in cytosine-phosphate-guanosine (CpG) dinucleotides to form 5-methylcytosine. DNA methylation, when it occurs within the CpG islands around the gene promoters or enhancers, is known to recruit transcriptional repressors and actively inhibit downstream gene transcription [98]. Paradoxically, transcriptional activation of genes is correlated with DNA hypomethylation [99]. In human and mouse osteoarthritic cartilage, destabilization of the medial meniscus was associated with PPARγ repression due to an aberrant surge in DNMT1/DNMT3a levels and subsequent hypermethylation of the PPARγ promoter [100]. Consequently, regulating the methylation status of the PPAR promoter in chondrocytes may decelerate OA progression. This line of reasoning has led to the emergence of many potential OA therapeutic drugs.

### 6.1. 5-Aza-2′-deoxycytidine (5Aza)

5Aza, a pharmacological DNA demethylating agent, is capable of reversing the hypermethylation of the PPAR promoter in osteoarthritic chondrocytes, thereby reinstating the expression of PPARγ. By inhibiting the expression of pro-inflammatory factors in osteoarthritic chondrocytes and augmenting the expression of antioxidant enzymes, 5Aza exerts protective effects. However, such cartilage-protective effects of 5Aza were found to be absent in PPARγ knockout mice, suggesting that 5Aza primarily confers protection to osteoarthritic cartilage by reestablishing PPARγ expression in osteoarthritic chondrocytes [100].

### 6.2. Diacerein

Chen and colleagues have identified a novel therapeutic potential of diacerein, a symptomatic slow-acting drug utilized in OA treatment [101,102]. Known for its analgesic, anti-inflammatory, and anti-catabolic properties coupled with a favorable safety profile, diacerein also inhibits IL-1 signaling [103,104]. The investigators discovered that diacerein significantly impedes the upregulation of DNMT1/3a, hypermethylation of the PPARγ promoter, and PPARγ depletion, thereby attenuating cartilage damage and effectively rectifying the expression of antioxidant enzymes and inflammatory cytokines [102].

### 6.3. The Active Constituents of Dabushen Decoction

The Dabushen decoction (DD), an ancient traditional Chinese medicinal formulation, comprises seven traditional Chinese medicines, including *Rehmanniae radix praeparata*,* Lophatheri herba*,* Schisandrae chinensis fructus*,* Zingiberis rhizoma*,* Cinnamomi ramulus*,* Alismatis rhizoma*,* and Glycyrrhizae radix et rhizoma*. It contains five active ingredients: alisol A, emodin, taxifolin, isoliquiritigenin, and schisandra C, which have been found to alleviate OA by specifically targeting DNMT1 to safeguard PPARγ [90]. 

## 7. Association between PPARα/γ and OA In Vivo

The targeted activation of PPARα/γ has been demonstrated to mitigate disease progression in OA models of animals induced via meniscal instability. Table 4 encapsulates the relevant studies highlighting the impacts of PPAR agonists on OA progression in vivo.

In one study, a partial medial meniscectomy was performed on the right knee joint of guinea pigs to establish an OA model. The osteoarthritic guinea pigs were subsequently administered 20 mg/kg/day of the PPAR agonist pioglitazone. The results indicated a significant reduction in tibial plateau cartilage defects both visually and histologically in the treatment group [105]. In another study by Boileau et al., an OA model was established by performing anterior cruciate ligament section surgery on dogs, followed by oral administration of either 15 mg/day or 30 mg/day of pioglitazone over 8 weeks. The results evidenced that pioglitazone attenuated cartilage damage in a dose-dependent manner [106]. Further studies revealed that in male osteoarthritic mice with unstable menisci, a joint cavity injection of the PPARα agonist Wy-14643 at a dosage of 5 mg/kg led to a notable reduction in articular cartilage destruction after 4 weeks [40]. In another OA mouse study, researchers discovered that losartan could hinder OA progression by elevating PPARγ expression [88].

The targeted activation of PPARγ has been found to decelerate AGEs-induced disease progression in OA models. AGEs overproduction and accumulation in vivo is a well-documented initiator and progressor of OA lesions [108,109]. In one experiment, different concentrations of d-ribose were injected into the knee joint cavity of rabbits, and the rabbits were subsequently made to run several hundred meters daily to simulate an age-related OA model. Following this, the rabbits were orally administered the PPARγ agonist pioglitazone. The findings suggested that pioglitazone has the potential to alleviate the severity of AGEs-induced OA in rabbits [107]. A similar methodology was employed in another study where AGE-BSA was injected into the joint cavity of male mice to create an age-related OA model. After oral administration of pioglitazone, the samples were collected 8 weeks later. Consistent with the previous findings, pioglitazone was found to reverse AGEs-induced cartilage degeneration and apoptosis in a mouse model [45].

The targeted activation of PPARγ has also been shown to alleviate joint inflammation and cartilage damage induced by high glucose levels. OA has been proposed to be positively associated with glucose imbalance, metabolic dysfunction, and diabetes mellitus (DM) [110,111,112]. Epidemiological studies have indicated an independent role of diabetes in the development and progression of OA [113,114,115,116]. In an experiment, streptozotocin was injected into the peritoneal cavity of 4-week-old male ICR mice to induce a hyperglycemia mouse model. The mice were then orally administered 10 mg/kg/day of pioglitazone, and the samples were collected for analysis 4 weeks later. The results indicated that hyperglycemia-induced cartilage damage in diabetic mice was effectively reversed via pioglitazone [44].

## 8. Conclusions and Prospects

OA is a prevalent degenerative joint disease, hallmarked by progressive cartilage degradation, synovitis, and subchondral bone remodeling. Despite the current treatment strategies for OA being restricted, PPAR, a ligand-activated transcription factor and a constituent of the nuclear receptor superfamily, has surfaced as a prospective target for OA therapeutics. PPAR is subdivided into three subtypes—PPARα, PPARγ, and PPARβ/δ—and activation of PPARα/β has been demonstrated to mitigate OA via various pathways. The targeted activation of PPARα/β has the ability to inhibit the synthesis of MMPs in chondrocytes, diminish the expression of inflammatory mediators such as NO and PGE2, regulate the autophagy activity of chondrocytes, and suppress chondrocyte apoptosis. Moreover, PPARγ can be activated by an array of small molecules, including abietic acid, astragalin, betulinic acid, mangiferin, oridonin, and losartan, which can inhibit the catabolism of chondrocytes. In addition, substances like 5Aza, diacerein, and active constituents of the Dabushen decoction can manipulate PPARγ promoter methylation to sustain PPARγ content in OA chondrocytes, thereby decelerating OA progression. Mechanistically, PPARα/γ agonists or active small molecules that activate PPARα/γ can inhibit the inflammatory response in OA cartilage by modulating the NF-κB signaling pathway, regulating chondrocyte autophagy activity via the Akt/mTOR signaling pathway, and controlling chondrocyte apoptosis by adjusting the MAPK signaling pathway. At the animal level, the targeted activation of PPARα/γ has been evidenced to exert a protective effect on cartilage under conditions of trauma, AGEs, or high glucose stimulation. Therefore, the targeted activation of PPARα/γ might represent a promising strategy for OA alleviation.

Although the targeted activation of PPARα/γ has demonstrated promising results in OA mitigation, additional research is required to fully elucidate the mechanisms involved. A potential research direction could be the regulation of lipid metabolism via PPARα, which might also contribute to cartilage protection. Fibrates, which are clinically utilized lipid-lowering drugs and PPARα agonists, have been evidenced to suppress the expression of inflammatory mediators in chondrocytes and modulate their autophagy activity [41]. Furthermore, research into ferroptosis, a novel form of iron-dependent programmed cell death, has indicated that lipid peroxidation of chondrocyte membranes induced via iron overload can precipitate chondrocyte death. Future research could explore whether PPARα agonists, like fenofibrate, can alleviate OA by inhibiting ferroptosis in chondrocytes. In addition, drug delivery systems, such as nanoparticles or hydrogels, could be amalgamated with PPAR agonists to facilitate sustained release and effectively protect cartilage over an extended duration.

## Figures and Tables

**Table 1:** PPAR subtypes, chromosome localization, natural ligands, synthetic ligands, and biological effects.

Subtype	Chromosome	Site of Expression	Natural Ligands	Synthetic Ligands	Biological Effects
PPARα	22	Liver, heart, skeletal muscles, BAT, intestine, kidneys, cartilage	Palmitic acid Palmitoleic acid Oleic acid Linoleic acid Stearic acid Pristanic acid Arachidonic acid Eicosatetraenoic acid Leukotriene B4	Fenofibrate Bezafibrate Gemfibrozil Wy-14643	fatty acid uptake and oxidation inflammation vascular function
PPARγ	3	WAT, liver, skeletal muscles, brain, stomach intestine, immune cells	Linoleic acid Arachidonic acid Eicosatetraenoic acid PGJ2, Linoleic acid 9-HODE 13-HODE	Troglitazone Pioglitazone Rosiglitazone BRL49653 GW1929	fatty acid uptake and storage inflammation glucose homeostasis
PPARβ/δ	6	Ubiquitous	FA Retinoic acid Carbaprostacyclin	GW501516 GW0742 GW501516	fatty acid metabolism inflammation macrophage lipid homeostasis

WAT, white adipose tissue; BAT, brown adipose tissue; PGJ2, Prostaglandin J2; 9-HODE, 9-hydroxyoctadecadienoic acid; 13-HODE, 9-hydroxyoctadecadienoic acid; FA, Fatty acids.

**Table 2:** The effect of PPARα/γ agonists on chondrocytes.

Isotype of PPAR	Therapeutic Agent	Author (Year)	Subjects	Findings
PPARα	Wy-14643 (PPARα agonist)	Clockaerts et al., 2011 [39]	Human OA cartilage explants	Inhibited the inflammatory and destructive responses
PPARα	Wy-14643 (PPARα agonist)	Zhou et al., 2018 [40]	Mouse chondrocytes	Promoted proteoglycan synthesis via autophagy enhancement in OA chondrocytes concomiant with the elevation of Akt and ERK phosphorylation
PPARα	Fenofibrate (PPARα agonist)	Nogueira-Recalde et al., 2019 [41]	Ageing human and OA Chondrocytes	Protect against cartilage degeneration seen with ageing and OA targeting lipid metabolism
PPARγ	15d-PGJ2 and GI262570 (PPARγ agonist)	Sabatini et al., 2002 [42]	Rat cartilage	Inhibit cytokine-induced proteoglycan degradation mediated by both aggrecanase and MMPs
PPARγ	Pioglitazone (PPARγ agonist)	Chen et al., 2014 [43]	Rabbit chondrocytes	Modulates TNF-α and MMP-13 expression in cultured rabbit chondrocytes via NF-κB signaling
PPARγ	Pioglitazone (PPARγ agonist)	Chen et al., 2015 [44]	Human chondrocytes	Ameliorate hyperglyce mia-induced inflammatory responses and collagen degradation
PPARγ	Pioglitazone (PPARγ agonist)	Zhang et al., 2016 [45]	Human chondrocytes	Inhibits AGEs-induced MMPs and apoptosis by suppressing the activation of MAPK and NF-κB
PPARγ	Pioglitazone (PPARγ agonist)	Ma et al., 2015 [46]	Human chondrocytes	Inhibit the effects of AGEs-induced inflammatory response
PPARγ	Pioglitazone (PPARγ agonist)	Wang et al., 2018 [47]	Human chondrocytes	Maintains cell viability by activating the Akt/mTOR signaling pathway as well as inducing chondrocyte autophagy
PPARγ	GW1929 (PPARγ agonist)	Ni et al., 2021 [48]	Rat chondrocyte	Attenuates IL-1β-induced cell apoptosis by inhibiting NOX2/ ROS/p38MAPK activation

**Table 3:** The effect of active small molecules activating PPARγ on chondrocytes.

Isotype of PPAR	Therapeutic Agent	Author (Year)	Subjects	Findings
PPARγ	Ozone	Sun et al., 2022 [76]	Rat chondrocytes	Improved autophagy via activating PPARγ/ mTOR signaling and suppressing inflammation in chondrocytes treated with IL-1β
PPARγ	Abietic acid	Kang et al., 2018 [83]	Human OA chondrocytes	Suppressed IL-1β-induced inflammation in human OA chondrocytes
PPARγ	Astragalin	Ma et al., 2015 [84]	Human OA chondrocytes	Suppressed IL-1β-induced inflammatory mediators; inhibited IL-1β-induced NF-κB and MAPK activation
PPARγ	Betulinic acid	Wang et al., 2015 [85]	Human OA chondrocytes	Inhibited IL-1β-induced inflammation
PPARγ	Mangiferin	Qu et al., 2017 [86]	Human OA chondrocytes	Inhibits IL-1β-induced inflammatory response
PPARγ	Oridonin	Jia et al., 2019 [87]	Human OA chondrocytes	Inhibits IL-1β-induced inflammatory response
PPARγ	Losartan	Deng et al., 2021 [88]	Human OA chondrocytes	Arrest the progression of OA via upregulating PPARγ expression and inactivating the TGF-β1 signaling pathway
PPARγ	Oleanolic acid	Kang et al., 2017 [89]	Primary mouse articular chondrocytes	Prevented the high glucose-induced cell injury
PPARγ	Dabushen decoction	Qiu et al., 2022 [90]	Rat chondrocytes	Ameliorate IL-1β-induced downregulation of COL II and the production of MMP-13

**Table 4:** The effect of PPARα/γ on OA experimental animals.

Isotype of PPAR	Therapeutic Agent	Author (Year)	Subjects	Findings
PPARα	Wy-14643 (PPARα agonist)	Zhou et al., 2018 [40]	DMM OA mice	Promoted proteoglycan synthesis via autophagy enhancement in OA chondrocytes concomitant with the elevation of Akt and ERK phosphorylation
PPARγ	Pioglitazone (PPARγ agonist)	Chen et al., 2015 [44]	Diabetic Mice	Show chondroprotection on mouse cartilage damage in diabetic mice
PPARγ	Pioglitazone (PPARγ agonist)	Zhang et al., 2016 [45]	AGEs-induced OA mice	Inhibiting the apoptosis and cartilage degradation
PPARγ	Losartan	Deng et al., 2021 [88]	DMM OA mice	Alleviates OA in DMM mice via PPARγ -mediated inactivation of the TGF-β1/Smad 2/3 signaling pathway
PPARγ	The active constituents dabushen decoction	Qiu et al., 2022 [90]	Papain with L-cysteine-induced OA rats	Ameliorate OA via PPARγ preservation by targeting DNMT1
PPARγ	DNA demethyla- ting agent 5Aza (5-Aza-2′-deoxyc ytidine)	Zhu et al., 2019 [100]	Wild type C57BL/6 mice; PPARγ knockout mice	PPARγ presservation via promoter demethylation alleviates OA in mice
PPARγ	Diacerein	Chen et al., 2022 [102]	DMM OA mice	Alleviates oxidative stress and OA in mice by reversing epigenetic PPARγ suppression
PPARγ	Pioglitazone (PPARγ agonist)	Kobayashi et al., 2005 [105]	Guinea pigs	Reduce the progression of experimental OA in guinea pigs
PPARγ	Pioglitazone (PPARγ agonist)	Boileau et al., 2017 [106]	ACLT dogs	Inhibit major signaling pathways of inflammation and reduce the synthesis of cartilage catabolic factors responsible for articular cartilage degradation
PPARγ	Pioglitazone (PPARγ agonist)	Li et al., 2016 [107]	Rabbit OA model	Reduce the severity of the AGEs-induced OA in a rabbit model

## Data Availability

Not applicable.
